# Effect of Chlorogenic Acid (5-Caffeoylquinic Acid) Isolated from *Baccharis oxyodonta* on the Structure and Pharmacological Activities of Secretory Phospholipase A2 from *Crotalus durissus terrificus*

**DOI:** 10.1155/2014/726585

**Published:** 2014-09-02

**Authors:** Daniela O. Toyama, Marcelo J. P. Ferreira, Paulete Romoff, Oriana A. Fávero, Henrique H. Gaeta, Marcos H. Toyama

**Affiliations:** ^1^UNESP, Campus Experimental do Litoral Paulista, PraÇa Infante Dom Henrique s/no, Bairro Parque Bitaru, 11330-900 São Vicente, SP, Brazil; ^2^Departamento de Botânica, Instituto de Biociências, Universidade de São Paulo, RuadoMatão 277, 05508-090 São Paulo, SP, Brazil; ^3^Escola de Engenharia, Curso de Química, Universidade Presbiteriana Mackenzie, Rua da Consolação 930, 01302-907 São Paulo, SP, Brazil; ^4^Centro de Ciências Biológicas e da Saúde, Curso de Ciências Biológicas, Universidade Presbiteriana Mackenzie, Rua da Consolação 930, 01302-907 São Paulo, SP, Brazil

## Abstract

The aim of this paper was to investigate the effect of chlorogenic acid (5-caffeoylquinic acid, 5CQA), isolated from* Baccharis oxyodonta*, on the structure and pharmacological effect of secretory phospholipase A2 (sPLA2) from* Crotalus durissus terrificus*. All* in vitro* and* in vivo* experiments were conducted using a purified sPLA2 compared under the same experimental conditions with sPLA2 : 5CQA. 5CQA induced several discrete modifications in the secondary structure and the hydrophobic characteristics of native sPLA2 that induced slight changes in the α-helical content, increase in the random coil structure, and decrease of fluorescence of native sPLA2. Moreover, 5CQA significantly decreased the enzymatic activity and the oedema and myonecrosis induced by native sPLA2. As the catalytic activity of sPLA2 plays an important role in several of its biological and pharmacological properties, antibacterial activity was used to confirm the decrease in its enzymatic activity by 5CQA, which induced massive bacterial cell destruction. We found that 5CQA specifically abolished the enzymatic activity of sPLA2 and induced discrete protein unfolding that mainly involved the pharmacological site of sPLA2. These results showed the potential application of 5CQA in the snake poisoning treatment and modulation of the pathological effect of inflammation induced by secretory PLA2.

## 1. Introduction

At present, phospholipase A2 (PLA2) (EC 3.1.1.4) can be classified into various groups and subgroups according to a complex molecular taxonomy. Several groups of PLA2 have recently been isolated and characterized. One of the most investigated groups of PLA2 includes secretory PLA2 (sPLA2), which is primarily found in the venom of several animals. sPLA2 exhibits well-established functions in the digestion of dietary phospholipids, which have important functions in the host's defence against bacterial infections and are involved in pathological processes, such as atherosclerosis and cancer [1, 2]. Moreover, mammalian genomes encode several types of sPLA2-binding proteins, indicating that sPLA2 may have enzyme-independent activities related to the binding for specific sPLA2 receptor on the target cells [3].

Several recent studies have shown that snake venom secretory PLA2 (SVsPLA2) as well as mammalian secretory phospholipase A2 (msPLA2) have similar mechanism of action [4, 5]. In* in vitro* studies, secretory PLA2 from human can be induce similar pharmacological events to those of snake venom secretory PLA2 [6]. Because the similarity in the structure, function and pharmacological effects between human secretory phospholipase A2 and snake venom secretory phospholipase A2, snake venom secretory phospholipase A2 has been used for molecular target to evaluate the anti-inflammatory effects of natural products and other drugs. In these studies, the focus is the ability of these compounds to decrease or inhibited the arachnoid acid synthesis [7–9]. This approach could especially be useful for developing of the new drugs to control of Arachidonic acid production during the inflammatory disease [10–12]. In this regard, the search for new molecules that can significantly reduce the enzymatic activity of sPLA2 and decrease the production of arachidonic acid through this route is very important from a therapeutic standpoint [13, 14].

Various natural compounds have the potential to inhibit or negatively modulate the activities of PLA2s and other enzymes involved in the cascade of arachidonic acid, offering a potential method for reducing and controlling the inflammatory process. Chlorogenic acid derivatives (CGAs) are esters formed between cinnamic acid derivatives and quinic acid and they are one of the most important groups of phenolic secondary metabolites produced by certain plant species. These compounds play a major role in the plant's response to various biotic and abiotic stresses [15–17]. Additionally, CGAs are one of the most abundant polyphenols in the human diet and they display several important roles in the therapeutic properties of many plant extracts, such as antioxidant activity [18–21].

Recently, Chagas-Paula et al. [22] showed that chlorogenic acids from* Tithonia diversifolia* have a better anti-inflammatory effect than indomethacin. Therefore, the aim of this paper was to evaluate the ability of chlorogenic acid (5-caffeoylquinic acid, 5CQA) to modulate the pharmacological and structural properties of sPLA2 isolated from the Brazilian rattlesnake* Crotalus durissus terrificus*.

## 2. Material and Methods

### 2.1. Materials

The venom from* Crotalus durissus terrificus* (*C. d. terrificus*) was kindly donated by the Butantan Institute (São Paulo, Brazil). The solvents, chemicals, and reagents used for protein purification and characterisation (HPLC grade or higher) were acquired from Sigma-Aldrich Chemicals (Spruce St., St. Louis, United State), Merck (Whitehouse Station, NJ, United State), and Bio-Rad (Hercules, CA, United State). Male Swiss mice (20–25 g) were obtained from the Multidisciplinary Centre for Biological Research (CEMIB) of the State University of Campinas (UNICAMP). The animals were maintained under standard conditions (22 ± 2°C; 12 h light/dark cycle) with food and water available* ad libitum*. All animal experiments were performed in accordance with Brazilian laws for the Care and Use of Laboratory Animals, and the protocols were approved by the Committee of Ethics from UNICAMP number 2898-1.

### 2.2. Extraction and Purification of Chlorogenic Acid (5-Caffeoylquinic Acid, 5CQA)

Aerial parts (twigs and leaves) of* Baccharis oxyodonta* DC. were collected in Campos do Jordão, São Paulo, SP, in August 2010 (flowering plant). The voucher specimen was deposited at Dom Bento Pickel Herbarium of Instituto Florestal de São Paulo under number SPSF8981.

**Figure 1:**
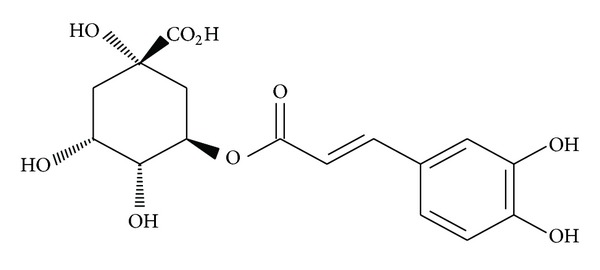
Chemical structure of chlorogenic acid (5-caffeoylquinic acid, 5CQA).

Dried and powdered aerial parts (1110 g) were defatted with* n*-hexane and subsequently extracted with methanol (MeOH) at room temperature. The crude methanolic extract (128.05 g) was suspended in MeOH : H_2_O (1 : 2 v/v) and successively partitioned with hexane, dichloromethane (CH_2_Cl_2_), ethyl acetate (EtOAc), and* n*-butanol (*n*-BuOH). The EtOAc phase was subjected to column chromatography over Sephadex LH-20 and eluted with MeOH to yield 12 fractions (A1–A12). The A3 fraction (50.5 mg) was subjected to high performance liquid chromatography (HPLC) purification, identified through ^1^H and ^13^C NMR spectra, and compared with the literature data [23] as 5-O-(*E*)-caffeoylquinic acid (chlorogenic acid, 5CQA), as shown in Figure 1.

### 2.3. Purification of Phospholipase A2

To purify the* C. d. terrificus* sPLA2, whole venom was first fractionated as previously described [24]. Dried venom (45 mg) was dissolved in ammonium bicarbonate buffer (0.2 M, pH 8.0) and clarified by centrifugation (4,500 ×g, 1 min). The supernatant was injected into a molecular exclusion HPLC column (Superdex 75, 1 × 60 cm, Pharmacia), and the chromatographic run was performed with a flow rate of 0.2 mL/min for the elution of fractions. The absorbance was monitored at 280 nm. The separated crotoxin-like fraction was immediately lyophilised. The lyophilised fraction was then subjected to reverse-phase chromatography using a *μ*-Bondapack C18 column (0.39 × 30 cm) coupled to an analytical HPLC system (LC-2000Plu, Jasco Corp., Japan) with a flow rate of 1 mL/min for fraction elution. The absorbance was monitored at 280 nm. Afterwards, this fraction was eluted using a nonlinear gradient with buffer A (0.1% trifluoroacetic acid [TFA] in Milli-Q water) and buffer B (66% acetonitrile in buffer A). The final fraction was the* C. d. terrificus* sPLA2, and its purity was evaluated by tricine SDS-PAGE and mass spectrometry on a MALDI-TOF mass spectrometer as previously described by Cotrim et al [25].

### 2.4. Treatment of sPLA2 with 5CQA

The incubations of* C. d. terrificus* sPLA2 with purified 5CQA at (mol : mol) were performed according to the described procedure [25]. 5CQA was dissolved in dimethylsulfoxide (DMSO). The final concentration of DMSO in the solution never exceeded 1% during incubation. An aliquot of the 5CQA solution (400 *μ*L of 0.1 mM solution) was added to 400 *μ*L of a homogenised, purified* C. d. terrificus* sPLA2 solution (1 mg/mL). The mixture was then incubated for 90 min at room temperature, and 200 *μ*L aliquots were loaded onto a preparative reverse-phase column to separate the treated enzyme (sPLA2 : 5CQA). Following column equilibration with HPLC buffer A (aqueous 0.1% TFA), the samples were eluted using a discontinuous gradient of HPLC buffer B (66.6% acetonitrile in 0.1% TFA) at a constant flow rate of 1.0 mL/min. The chromatographic run was monitored at 214 nm.

### 2.5. Circular Dichroism Spectroscopy

Secondary structure can be determined by circular dichroism (CD) spectroscopy in the “far-UV” spectral region (190–250 nm), and the CD spectrum of a protein in the “near-UV” spectral region (250–350 nm) is sensitive to certain aspects of the tertiary structure. In this study, we used both assay types to evaluate the secondary structure and monitor shifts in the tertiary structure of native sPLA2 and sPLA2s that were chemically modified by 5CQA. To determine the protein secondary structure, sPLA2 and sPLA2 : 5CQA were dissolved in 10 mM sodium phosphate buffer (pH 7.4), and the final protein concentrations were adjusted to 8.7 mM. This protein solution was then subjected to centrifugation, at 4000 ×g for 5 min, and the resulting supernatant was transferred to a 1 mm path-length quartz cuvette. CD spectra within a wavelength range of 185–300 nm were acquired in-house with a J720 spectropolarimeter (Jasco Corp., Japan) using a bandwidth of 1 nm and a response time of 1 s. Data collection was performed at room temperature with a scanning speed of 100 nm/min. Nine scans were obtained for each sample, and all spectra were corrected by subtracting buffer blanks. The near-UV CD spectrum (>250 nm) of the samples provided information on the tertiary protein structure. The signals obtained in the range of 250–300 nm were caused by the absorption, dipole orientation, and nature of the surrounding environment around the phenylalanine, tyrosine, cysteine (or S-S disulphide bridges), and tryptophan residues in the protein. In this study, the CD HPLC detector from Jasco Corp., Japan, was used to enable scanning of the sPLA2 and sPLA2 : 5CQA peaks.

### 2.6. Mass Spectrometry

The molecular masses of sPLA2 and sPLA2 : 5CQA were determined by matrix-assisted laser desorption ionisation-time-of-flight mass spectrometry (MALDI-TOF) using a Voyager-DE PRO MALDI-TOF mass spectrometer (Applied Biosystems). One microliter of sample (sPLA2 and sPLA2 : 5CQA) with a concentration adjusted to 3 mg/mL in 0.1% TFA was mixed with 2 *μ*L of the matrix *α*-cyano-4-hydroxycinnamic acid, 50% acetonitrile, and 0.1% TFA (v/v). The matrix was prepared with 30% acetonitrile and 0.1% TFA (v/v). Ion masses were determined with an acceleration voltage of 25 kV; the laser was operated at 2890 kJ/com^2^ with a 300 ns delay and the linear analysis mode.

### 2.7. Enzymatic Assay of sPLA2

The sPLA2 activity was measured by following a previously described protocol [26] for a 96-well plate assay using 4-nitro-3-octanoyloxy-benzoic acid (4N3OBA or NOBA; Enzo Life Sciences, Inc. Farmingdale, NY, USA) as the substrate. Enzyme activity, which was expressed as the initial velocity of the reaction (Vo), was calculated on the basis of the increase in absorbance after 20 min. All assays were performed using a sample size of 12, and the absorbance at 425 nm was measured by using a SpectraMax 340 multiwell plate reader (Molecular Devices, Sunnyvale, CA). After the addition of sPLA2 (20 *μ*g), the reaction mixture was incubated for 40 min at 37°C, and the absorbance was read at 10 min intervals. The effect of the substrate concentration on enzyme activity was determined by measuring the absorbance increase after a 20 min incubation in Tris-HCl buffer, pH 8.0, at 37°C. All assays were performed in triplicate, and the absorbance at 425 nm was measured by using a SpectraMax 340 multiwell plate reader (Molecular Devices, Sunnyvale, CA, USA). The remaining enzymatic assay was conducted as described above. 5CA was dissolved in 1% DMSO.

### 2.8. Antibacterial Activity

The* Clavibacter michiganensis pv michiganensis* (gram-positive) phy bacterial strain was collected from fresh agar plates and suspended in distilled sterilized water (A650 nm = 3 × 10^8^ CFU/mL). Aliquots of the bacterial suspension were diluted to 10^3^ CFU/mL and incubated with sPLA2 or sPLA2 : 5CQA (150 *μ*g/mL) for 1 h at 37°C. After incubation, survival was assayed on nutrient (Difco) plates (*n* = 5). Bacterial viability was tested by CFU counting.

### 2.9. Scanning Electron Microscopy of Bacteria (*Clavibacter michiganensis pv michiganensis*)

Samples were collected for examination after the incubation time necessary to induce antimicrobial activity (60 min). After centrifugation, the pellets were fixed at 48°C in 0.1 M cacodylate buffer (pH 7.4) containing 2.5% (v/v) glutaraldehyde for 12 h. In the second fixation, bacterial samples were fixed once more with 1% osmium tetroxide for 2 h at 48°C. The samples were then dehydrated in increasing concentrations of ethanol. The specimens were coated with gold in a vacuum using a sputter coater (BALZERS SCD 050). Electron micrographs were obtained using a JSM-5800LV-JEOL scanning electron microscope.

### 2.10. Paw Oedema

A paw oedema assay was performed using a previously described protocol [8]. Male Swiss mice (21 g) were anaesthetised by inhaling halothane. Posterior paw oedema was induced by the single subplantar injection of sPLA2 or 5CQA-pretreated sPLA2. Each site received an injection of 20 microlitres of sample (0.5 mg/mL); therefore, each site received 10 *μ*g per paw of the native sPLA2 or 5CQA-pretreated sPLA2. The paw volumes were measured immediately before the injection and at selected time intervals thereafter (0, 30, 60, 120, and 240 minutes) with a hydro-plethysmometer (model 7150, Ugo Basile, Italy). All drugs were dissolved in a 0.9% sterile saline solution. The results are expressed as the increase in paw volume (mL) calculated by subtracting the initial volume. The area under the time-course curve was also calculated (trapezoidal rule), and the results were expressed as the total oedema volume (millilitres per paw).

### 2.11. Evaluation of Myonecrosis

The liberation of creatine kinase (CK) from damaged muscle cells was determined by recording the enzyme activity in mouse plasma using the CK-NAc kit (Laborlab) as previously described [25]. The samples of native sPLA2 and sPLA2 : 5CQA were injected into the left gastrocnemius muscle of male Swiss mice (18–20 g; *n* = 5). The right gastrocnemius muscle was injected with 50 *μ*L of a 0.5 mg/mL sPLA2 sample. Control mice received an equal volume of 0.15 M NaCl. After 3 h, the mice were anaesthetised, and blood was collected from the abdominal* vena cava* into tubes containing heparin as an anticoagulant. The plasma was stored at 4°C for a maximum of 12 h before the assay. The level of CK was then determined with 40 *μ*L of plasma, which was incubated for 3 minutes at 37°C with 1.0 mL of the reagent according to the kit protocol. The resulting activity was expressed in U/L.

### 2.12. Statistical Analysis

The results are reported as the means ± SEM of replicated experiments. The significance of the differences between the means was assessed by an analysis of variance followed by Dunnett's test when several experimental groups were compared with the control group. The confidence limit for significance was 5%.

## 3. Results

**Figure 2:**
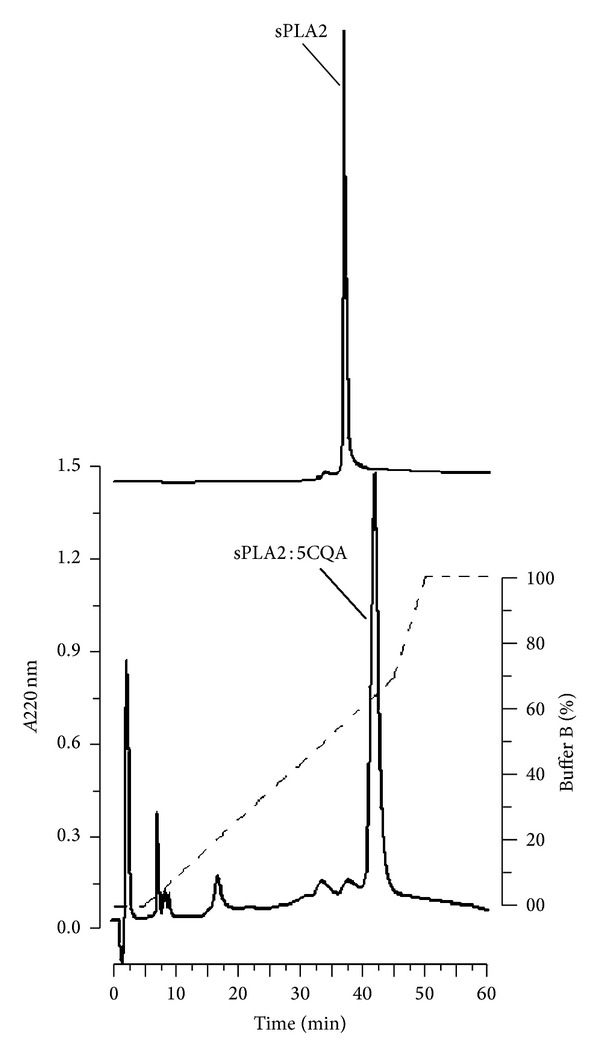
Purification and chemical modification of secretory phospholipase A2 (sPLA2). Fractionation of whole venom was performed by reverse-phase HPLC (C5 column 0.10 cm × 25 cm) using a nonlinear concentration gradient of buffer to obtain a high purity protein. This protein was designated as native sPLA2, which eluted at was eluted with a retention time of 35.8 min. sPLA2 chemically treated with 5-caffeoylquinic acid (5CQA) was subjected to HPLC purification for purification of sPLA2 : 5CQA complex, which it was was eluted at 40.3 min under the same chromatography conditions.

For the purification of sPLA2 and incubation of sPLA2 with 5CQA (Figure 1), we used a highly purified sPLA2 isolated from* C. d. terrificus* venom through reverse-phase chromatography (Figure 2), with its enzymatic activity evaluated using 4N3OBA as a substrate. SDS-PAGE revealed the presence of one protein band with a molecular mass of 14 kDa, which corresponded to sPLA2. Figure 2 shows the chromatographic profile of the native sPLA2, which was analysed using reverse-phase HPLC; the native sPLA2 was eluted with a retention time of 35.8 min, whereas 5CQA-pretreated sPLA2 was eluted at 40.3 min under the same chromatography conditions. This difference indicates an interaction between sPLA2 and 5cQA, which changes the hydrophobicity of sPLA2. In addition, we observed that the shape of sPLA2 : 5CQA was enlarged compared with sPLA2 purified from* Crotalus durissus terrificus* snake venom.

**Figure 3:**
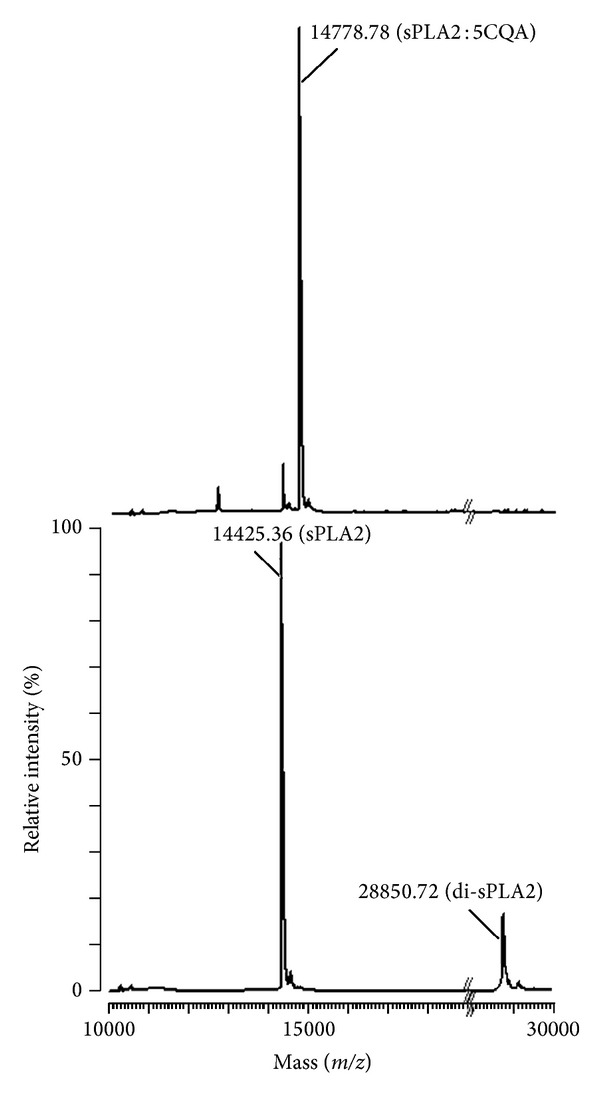
MALDI-TOF mass spectrometry analysis of native sPLA2 and sPLA2 : 5CQA shows a difference between the molecular masses, corresponding to one molecule of bound 5CQA. Additionally, this figure shows the mass of the sPLA2 dimer.

For the mass spectrometry, we analysed the native sPLA2 and 5CQA-pretreated sPLA2, and the results are shown in Figure 3. The native sPLA2 analysis showed the presence of native sPLA2 with molecular mass of 14,425.36 Da and its dimmer with molecular mass of 28,850.72 Da. The 5CQA-pretreated sPLA2 showed only one peak, with a molecular mass of 14,778.67 Da, which suggests that one molecule of 5CQA is bound to the protein structure, increasing the molecular mass of sPLA2 by 354.31 Da. The analysis is given in Figure 3.

**Figure 4:**
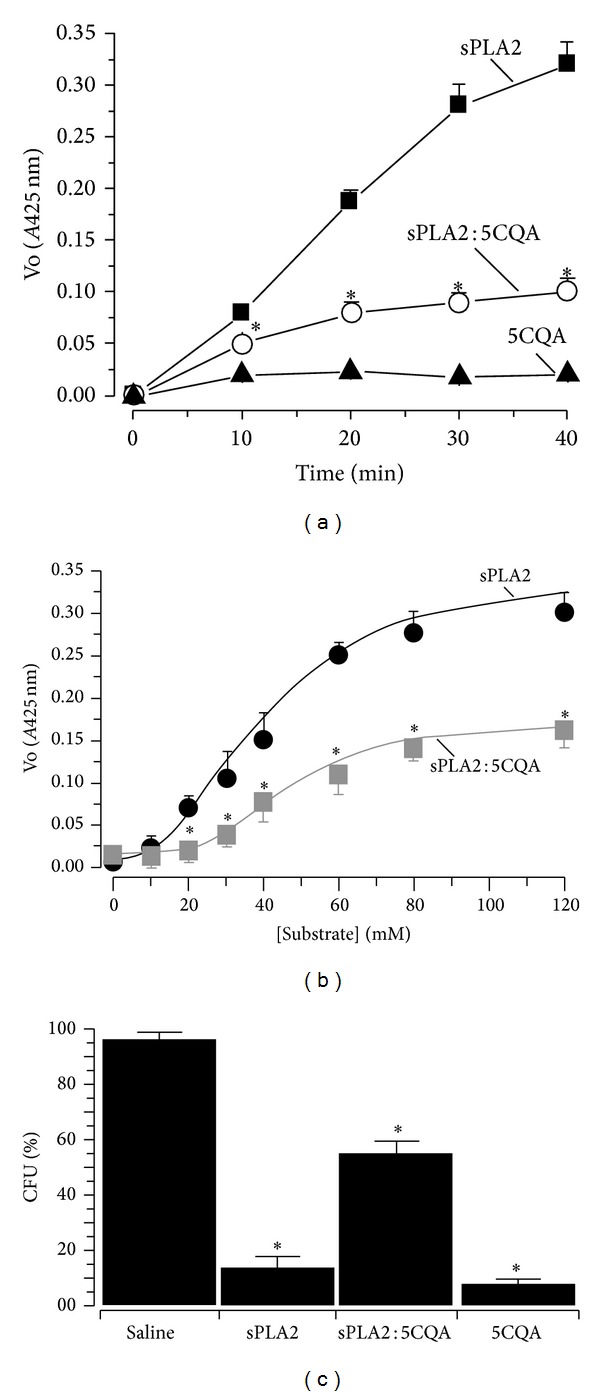
(a) Enzymatic activity was analysed using 4N3OBA as a substrate and monitored at a wavelength of 425 nm. sPLA2 : 5CQA shows a significant decrease compared with native sPLA2. (b) The effect of the substrate on the enzymatic activity of the native and 5CQA-pretreated sPLA2 (sPLA2 : 5CQA). Chemical treatment of sPLA2 with 5CQA shifts both the Km and Vmax of the native sPLA2. (c) The effect of native sPLA2 and sPLA2 : 5CQA against* Clavibacter michiganensis pv michiganensis *(gram-positive bacteria). Error bars indicate the SEM; *P < 0.05 compared with the saline control.

The effect of 5CQA on sPLA2 enzymatic activity was evaluated. The linear slope indicates that the rate of the enzymatic reaction and the increase in product formation is proportional to the enzyme reaction. As the reaction proceeds, the substrate is consumed and the acceleration decreases.  Figure 4(a) shows the time course effect of an enzymatic reaction. The native sPLA2 exhibited a linear rate increase over a 30 min reaction, and the sPLA2 : 5CQA showed a reduction in enzymatic activity of approximately 73 ± 8% over the same time period (Figure 4(a)). The sPLA2 of* Crotalus durissus terrificus* has been characterized as an allosteric enzyme in the presence of 4-nitro-3-octanoyloxy-benzoic acid, which is a chromogenic substrate that is specific for phospholipase A2 [24, 27], and the presence of the dimer structure of sPLA2 could be important for increasing the enzymatic activity of sPLA2. Based on the mass spectrometry results, which showed that the dimer of sPLA2 is not formed after the treatment with 5CQA, 5CQA can be inferred to inhibit the formation of the sPLA2 dimer and strongly decrease the enzymatic activity of native sPLA2. However, 5CQA may interact with the catalytic site of sPLA2 and strongly decrease its enzymatic activity, which is shown by effect of the substrate on the enzyme (Figure 4(b)).

**Figure 5:**
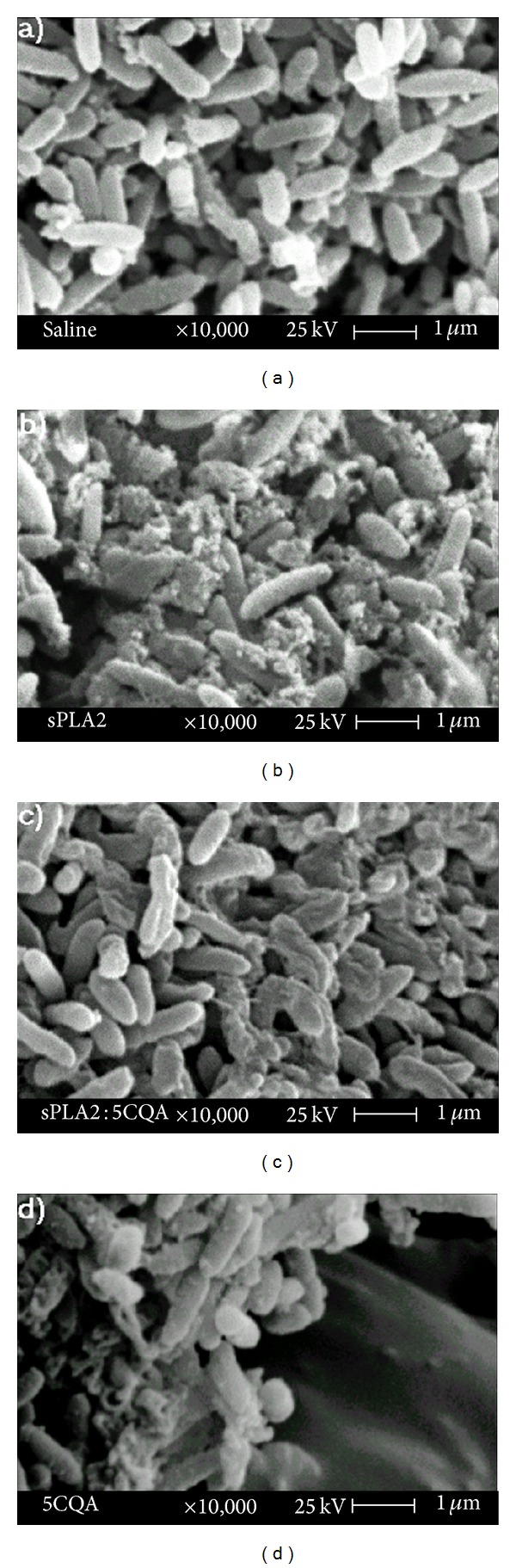
Antimicrobial effect of sPLA2 incubated with* Clavibacter michiganensis pv michiganensis* using a single dose of 150 *μ*g/mL for each sample. Scanning electron microscopy of* Clavibacter michiganensis pv michiganensis *in the absence of native sPLA2, designated as the control, (a) in the presence of sPLA2 and (b) in presence of sPLA2 : 5CQA and 5CQA.

Additionally, we analysed the correlations between the enzymatic activity of sPLA2 and its antibacterial activity. The assay was performed using* Clavibacter michiganensis michiganensis* (gram-positive). As shown in Figure 4(c), sPLA2 has a higher inhibitory potential on bacterial growth than 5CQA-pretreated sPLA2. Moreover, 5CQA was tested with an aliquot of the* Clavibacter michiganensis michiganensis*, revealing that 5CQA virtually abolished the bacterial growth rate (Figure 4(c)). Electron microscopy assessments of* Clavibacter michiganensis michiganensis* did not reveal superficial modifications in the absence of sPLA2 (Figure 5(a)). However, in the presence of sPLA2, we observed extensive vesiculation areas and pore formation on the bacterial membrane. These data suggest that the enzymatic activity of sPLA2 on bacterial membranes is crucial for the antibacterial activity of sPLA2 (Figure 5(b)). The results presented in Figure 5(c) show that the samples of 5CQA-pretreated sPLA2 induce the formation of vesicles on the surface of the bacterial membrane, which induces changes and deformation in the bacterial membrane. We also found that 5CQA induced extensive destruction of the bacterial membrane due to the formation of pores in the bacterial membrane, which appear to be more intense than the bacteria samples treated with sPLA2 (Figure 5(d)). The results for the antibacterial activity of the 5CQA-pretreated sPLA2 indicate that sPLA2 formed a stable complex with the 5CQA, confirming the results of mass spectrometry and resulting in the partial loss of the antimicrobial properties of native sPLA2 and 5CQA.

**Figure 6:**
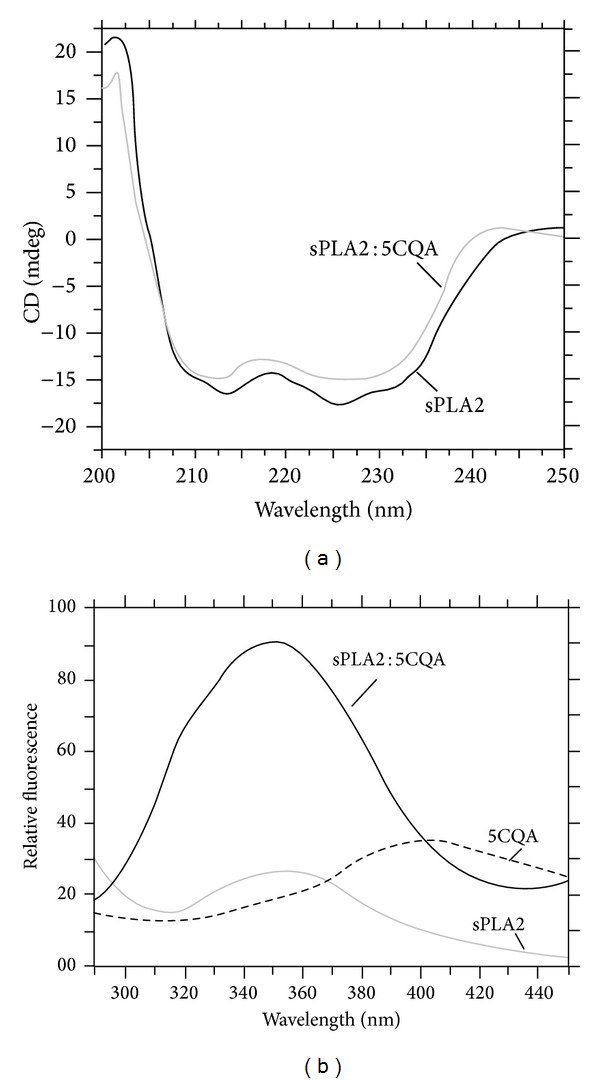
(a) CD spectra of native sPLA2 and sPLA2 : 5CQA. Data over the range of 185–280 nm are shown. The CD spectra are expressed in theta machine units in millidegrees. (b) Intrinsic fluorescence of native sPLA2, sPLA2 : 5CQA, and 5CQA measured with excitation at 280 nm and emission monitoring between 300 and 450 nm.

In Figure 6(a), few modifications were observed in the 270–280 nm wavelength region. However, some changes were observed in the CD and fluorescence spectra after the treatment with 5CQA. The CD spectra analysis showed modifications that were mainly in the regions corresponding to the *α*-helices, *β*-sheets, and random coils, suggesting that 5CQA can induce changes in the secondary structure of this enzyme (Figure 6(a)). The presence of aromatic amino acids, such as tryptophan and tyrosine, in the protein chain, allows for the use of fluorescence spectra, which are sensitive for the investigation of protein conformation and ligand binding.  Figure 6(b) shows an increase in the intensity of fluorescence emission spectra after treatment with 5CQA, suggesting that this phenolic derivative can change the structure of the protein at the tertiary structural level. The fluorescence spectrum of 5CQA showed that this compound has a peak of fluorescence near 400 nm. Therefore, the fluorescence increase of sPLA2 : 5CQA did not involve the intrinsic fluorescence spectra of 5CQA.

**Figure 7:**
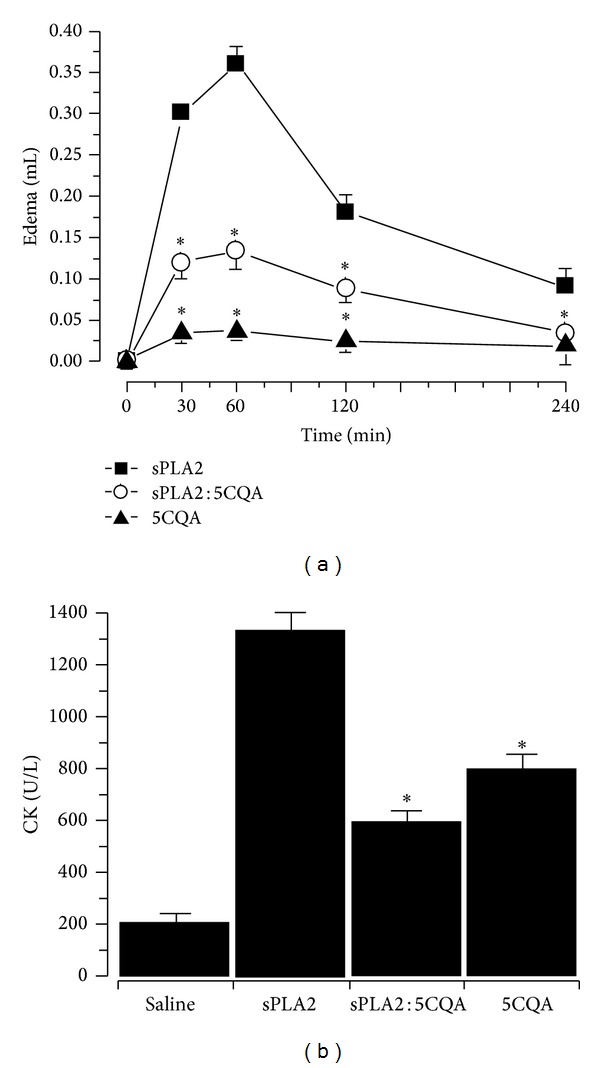
The results of the pharmacological assays. (a) Paw oedema induced after the injection of sPLA2 and sPLA2 : 5CQA (10 *μ*g/paw) into the right paw of Swiss mice. Measurements were performed after 30, 60, 120, 180, and 240 min, and no differences were observed after the treatment with 5CQA. (b) Myonecrosis was assayed based on the creatine kinase levels in Swiss mice. The right gastrocnemius muscle was injected with 50 *μ*L of a 0.5 mg/mL sPLA2 sample (native or 5CQA-treated). Control mice received an equal volume of 0.15 M NaCl. After 3 h, the mice were anaesthetised, and blood was collected from the abdominal vena cava into tubes containing heparin as an anticoagulant. The results are expressed as the units of enzymatic activity per litre (U/L). Error bars indicate the SEM. *P < 0.05 compared with sPLA2 activity.

As flavonoids can inhibit sPLA2 and consequently decrease its proinflammatory activity [26, 28, 29], the effect of 5CQA on sPLA2 was evaluated. Following subplantar injections of Swiss mice, sPLA2 had substantial potential to induce oedema after 60 min (Figure 7(a)). Under the same experimental conditions, 5CQA highly decreased the oedema effect induced by native sPLA2. The ability of sPLA2 to cause myonecrosis was also evaluated through the measurement of released CK.  Figure 7(b) shows that the intramuscular injection of sPLA2 and 5CQA induced an increase in plasma CK levels of 1375.79 ± 115.3 U/L and 789.65 ± 112.5 U/L, respectively, indicating their ability to cause muscle damage. 5CQA-pretreated sPLA2 significantly decreased the CK levels to 582.71 ± 91.7 U/L, a 57% decrease compared with the native sPLA2 (Figure 7(b)).

## 4. Discussion and Conclusion

In this study, sPLA2 from* C. d. terrificus *was modified by 5CQA, which is known for its anti-inflammatory activity. The chemical reaction of sPLA2 with quercetin or quercitrin resulted in the modification of the protein secondary structure as observed in CD assays and tryptophan fluorescent scanning [25, 26]. Although a secondary structure modification could be observed, the results do not allow for conclusions about the tertiary structure modification because the treatment of sPLA2 with 5CQA did not abolish the enzymatic and antibacterial activities or the oedema. We observed a significant decrease in the enzymatic velocity of the pharmacological activities. Therefore, 5CQA induced a secondary structure modification without leading to protein misfolding. Additionally, the enzymatic results suggested that enzyme inhibition did not involve the catalytic site; there were Km and Vmax changes compared with those of native sPLA2.

Toyama et al. [30] suggested that enzymatic activity is not required for the antibacterial activity or pharmacological effects induced by the* C. d. terrificus* PLA2 isoform, and similar results were observed by Diz Filho et al. [27] for* Crotalus durissus ruruima*. Cotrim et al. [25] demonstrated the correlation between the enzymatic and antibacterial activities against gram-positive bacteria, and our results strongly reinforce the ideas that the enzymatic activity of sPLA2 is crucial for this activity and that other molecular regions contribute to this effect. Native sPLA2 destroyed the bacterial membrane and induced membrane vesiculation, whereas sPLA2 treated with 5CQA induced only membrane vesiculation. Furthermore, our results showed that 5CQA has high antibacterial activity. Zhang et al. [31] suggested that 5CQA bound to the outer membrane causes disruption of the membrane, exhausting the intracellular potential, releasing cytoplasm macromolecules, and leading to cell death. These results reinforce the chemical interaction between sPLA2 and 5CQA.

5CQA, one of the most abundant polyphenols in the human diet, exerts potent anti-inflammatory, antibacterial, and antioxidant activities. The anti-inflammatory activity of 5CQA may involve multiple mechanisms of action, including the inhibition of the production and secretion of chemical mediators involved in the inflammatory process [32, 33]. Other studies also reported that 5CQA can inhibit the translocation of NFkB [32] and that the compound appears to be involved in the inactivation of various kinases. Zhao et al. [34] showed that 5CQA induced a significant reduction in IL-8 secretion, and Krakauer [35] demonstrated that 5CQA inhibited the secretion of several other cytokines including IL-1b. Azza et al. [36] showed that 5CQA induced both analgesic and anti-inflammatory properties by decreasing the levels of superoxide and peroxynitrite anion radicals, controlling the oxidative stress and, consequently, the inflammatory response. Therefore, at the present time, 5CQA induces its anti-inflammatory effect in three ways: inhibiting the production of or inducing the translocation of some chemical mediators involved in the inflammatory process or significantly decreasing the oxidative stress and, consequently, the inflammatory cascade.

We found that the anti-inflammatory effect of 5CQA involves the chemical interaction and structural modification induced by this compound; however, these modifications do not involve the catalytic unit of sPLA2. We also found that some pharmacological activities, such as inflammation and myonecrosis, were significantly decreased, but not abolished, after the treatment with 5CQA. This result is in agreement with the results in previously published papers [25, 27, 29, 30], which suggested that there are distinct pharmacological sites in the molecular region near the catalytic site involving the calcium binding loop [26], beta wing [37], and C-terminal [38] of sPLA2.

Therefore, we are the first to show that 5CQA leads to the inactivation of sPLA2, diminishing its enzymatic activity and binding to the sPLA2 receptor. In conclusion, the inhibition of sPLA2 by 5CQA showed a possible therapeutic application of this compound as a new candidate for developing drugs to treat inflammatory disease. This compound had a direct interaction with the sPLA2 protein and, consequently, significantly diminished the enzymatic, biological, and pharmacological effects that are involved in the toxic effects of rattlesnake venom. However, 5CQA has to be used correctly because the intramuscular application of this compound can induce myotoxicity, which can aggravate the patient's symptoms. There are several reports of adverse effects from the use of natural compounds, and many of these effects depend on the administration route of the compound [39].
